# Energy expenditure in caving

**DOI:** 10.1371/journal.pone.0170853

**Published:** 2017-02-03

**Authors:** Giorgia Antoni, Elisabetta Marini, Nicoletta Curreli, Valerio Tuveri, Ornella Comandini, Stefano Cabras, Silvia Gabba, Clelia Madeddu, Antonio Crisafulli, Andrea C. Rinaldi

**Affiliations:** 1 Gruppo Speleo Archeologico Giovanni Spano, Cagliari, Italy; 2 Department of Biomedical Sciences, University of Cagliari, Cagliari, Italy; 3 Department of Life and Environmental Sciences, University of Cagliari, Cagliari, Italy; 4 Corpo Nazionale del Soccorso Alpino e Speleologico, Sardinia, Italy; 5 Department of Mathematics and Informatics, University of Cagliari, Cagliari, Italy; 6 Department of Statistics, Universidad Carlos III de Madrid, Getafe, Spain; 7 Department of Medical Sciences, University of Cagliari, Cagliari, Italy; Vanderbilt University, UNITED STATES

## Abstract

The aim of this study was to determine the energy expenditure of a group of cavers of both genders and different ages and experience during a 10 hour subterranean exploration, using portable metabolimeters. The impact of caving activity on body composition and hydration were also assessed through bioelectrical impedance, and nutritional habits of cavers surveyed. During cave activity, measured total energy expenditure (TEE) was in the range 225–287 kcal/h for women-men (MET = 4.1), respectively; subjects had an energy intake from food in the range 1000–1200 kcal, thus inadequate to restore lost calories. Bayesian statistical analysis estimated the effect of predictive variables on TEE, revealing that experienced subjects had a 5% lower TEE than the less skilled ones and that women required a comparatively larger energy expenditure than men to perform the same task. BIVA (bioelectrical impedance vector analysis) showed that subjects were within the range of normal hydration before and after cave activity, but bioelectrical changes indicated a reduction of extracellular water in men, which might result in hypo-osmolal dehydration in the case of prolonged underground exercise. All these facts should be considered when planning cave explorations, preparing training programs for subjects practising caving, and optimizing a diet for cavers. Further, information gathered through this study could be of value to reduce accidents in caves related to increase in fatigue.

## Introduction

Caves are an hostile environment for human beings. Total darkness, high air humidity, muddy and slippery conditions, the recurring presence of rivers, waterfalls and/or lakes, characterise the underground world [1]. Temperature is usually constant within a cave, but can vary greatly in absolute terms, as caves are found in a variety of settings, from cold alpine environment to warm tropical rain forests. Notwithstanding these conditions, tens of thousands of cavers worldwide enjoy the exploration of natural cave systems as a recreational outdoor activity, although it certainly cannot be considered a mass sport [2]. Caves are abundant around the world. In Italy alone, some forty thousands caves are known [3]. In Britain, the main caving areas are the Mendip Hills, the Yorkshire Dales, and the Derbyshire Peak District, where limestone formations can be found [4]. The US are home to some of the most extensive cave systems in the word, including Mammoth Cave, with more than 400 miles explored [5].

Caving is a peculiar recreational activity, given the uniqueness of the environment where it takes place. The morphology of caves is very variable. Depths of several hundreds meters and kilometric lengths are not unusual, with explorations by cavers (also called spelunkers, potholers, or speleologists) lasting many hours, or even days, and requiring extensive walking, crawling, climbing and ropework, and considerable physical stamina [6].

Besides being the field for recreational cavers, caves have been also used as a natural laboratory where significant research activity is carried out by scientists of different disciplines, like geology, hydrogeology, and biology. Also, natural chambers, caverns and caves are sometimes found in mines. Cave systems have also been used as military transportation and escape routes and as weapons caches. Finally, showcase caves are visited by millions of tourists each year.

Despite this intense human frequentation of caves, however, very little is known about human behaviour in this austere environment. In particular, knowledge on physical activity and associated physiological processes during caving explorations is scant. The effects of high humidity, more commonly associated to elevated temperatures, on human exercise performance has been studied to some extent. In particular, it is well understood that dehydration (and hyperthermia), if sufficiently severe, will impair prolonged aerobic exercise performance, and that while acclimatization/adaptation might reduce the impact of high environmental temperatures, it provides limited protection when humidity is high [7–9]. However, given the particular mix of environmental conditions in the underground world, this knowledge cannot be transferred to exercise during cave exploration in a straightforward manner. As for caves, indeed, most of the available biomedical scientific literature deals with human circadian systems in caves [10] and with caving injuries [11–13], probably not surprisingly if one considers the medical and logistical challenges associated with cave rescue operations. Some specific studies have focused on very peculiar cases, such as the analysis of the cardio-vascular, neurological and metabolic physiological activities of cavers in the Naica Caves, Mexico, where temperature exceeds 45° C and humidity is well over 90% [14]. On the other hand, more limited attention has been devoted to the modification of biochemical and haematological parameters during conventional speleological practice [15–17].

In the attempt to contribute to fill this gap, we have measured the energy expenditure and impact of caving activity on body composition and hydration status of a group of cavers of both genders and different ages and experience during a 10 hour subterranean exploration. To this purpose, we have applied bioelectrical impedance vector analysis (BIVA) [18], a procedure usable in the field, that has demonstrated to accurately evaluate hydration status (classic BIVA) and body composition according to the two-compartment model (specific BIVA). Given the particular environment and the long-lasting sessions of caving it is possible to hypothesise that this kind of physical activity may lead to a physical stress which can be not trivial for the body homeostasis. In particular, we were interested in the potential impact of caving on hydration and energy expenditure. This knowledge would allow the design of specific training programs able to induce the specific adaptations required by caving, and to make informed recommendations regarding the proper nutrition and hydration of cavers during underground activity. Furthermore, information gathered through this study could be of value to reduce accidents in caves related to increase in fatigue.

## Materials and methods

### Participants

A total of forty subjects, 16 women and 24 men, were enrolled for this study. The study was performed in accordance with the Declaration of Helsinki and was approved by the Ethics Committee of the University of Cagliari. Written informed consent was obtained from all participants. They were all Italians, mean age (± standard deviation, SD) 44 ± 19 (range 25–63 years), and were categorized into three groups according to their caving experience: beginners (<5 years experience), amateurs (5–10 years experience) and experts (> 10 years experience). Before entering the study, subjects were carefully screened for cardiac, pulmonary, and metabolic problems, and for neurological diseases. None of them had any cardiovascular, respiratory, metabolic, or neurological disease, confirmed by clinical history, basal ECG and physical examination. None of the involved subjects has been taking any drug during the two weeks before cave exploration. Assessment of physical capacity was performed duringin the labor an incremental exercise test (IET) on cycle-ergometer (CUSTO Med, Ottobrunn, Germany). Gas exchange analysis was conducted with a gas analyzer (ULTIMA CPX, MedGraphics St. Paul, MN) calibrated immediately before each IET. Values anaerobic threshold (AT), maximum workload (W_max_) and maximum oxygen uptake (VO_2max_) were assessed. For all subjects, several anthropometric and nutritional status parameters were also measured, together with the hydration status before and after the cave exploration (see below). Cavers entered the cave in small groups (6–7 individuals) in different dates between June and September 2015. The underground exploration followed a common route for all subjects (total distance ≈ 3 Km), and began in the morning (10 a.m.) and ended in the evening of the same day (6:00–8:00 p.m.), lasting between 8 and 10 hours. While underground, all the subjects kept continuously on the move apart from short technical pauses (e.g., ropes and narrow passages) and a brief half-an-hour rest before beginning the way back toward the exit, and did not sleep.

### Cave description

**Fig 1:**
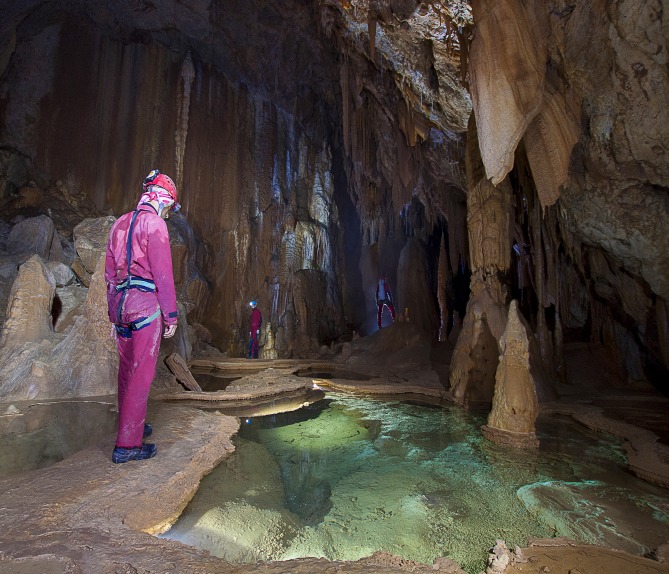
A snapshot of Su Palu cave, Gulf of Orosei, central-east Sardinia, Italy.

Field measurements were performed in the Su Palu cave, no. 1988 SA/OG of the Sardinian regional registry of caves [19], situated in the karst area of the Gulf of Orosei, central-east Sardinia, Italy (Fig 1). The cave is part, together with the Su Spiria-Monte Longos cave, of the Codula Ilune karst system extending for over 70 km, one of the largest in Europe [20,21]. Su Palu cave has been selected because of the size and proper combination of the elements which cavers usually encounter during their explorations, including pitches–that require complete personal equipment for progression on ropes–extensive sub-horizontal galleries, narrow passages, subterranean rivers and lakes. The internal temperature is constant year round ≈ 14–15°C, and cave air relative humidity is ≈ 95–100%, a normal value for caves with active waters [22].

### Measurement of underground energy expenditure

**Fig 2:**
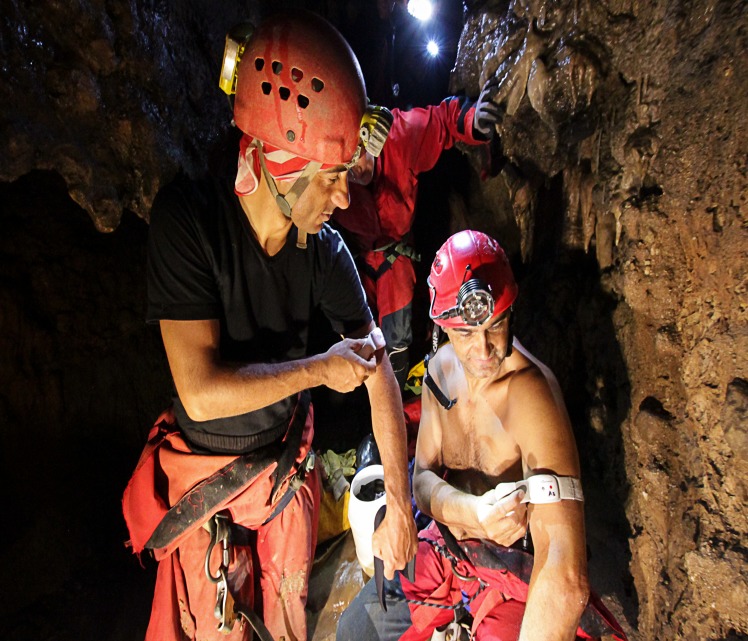
Cavers wearing armbands after a sump passage in the Su Palu cave (see main text for more details).

Energy expenditure was measured in 36 cavers (13 women, 23 men; age: 42.4 ± 8.5). Exclusion of four subjects from energy expenditure measurement was not due to any particular reason but availability of metabolimeters on the day of cave exploration. Anthropometric parameters and changes in bodycomposition and hydration status (see below) were assessed for all 40 cavers involved in the study. Metabolic parameters were assessed before, during and after caving activity by means of SenseWear Armband Fit Core (BodyMedia, Inc., Pittsburgh, PA), a wireless activity monitor which is equipped with sensors measuring skin temperature, heat flux, galvanic skin response and acceleration (sensor of movement) (Fig 2) [23,24]. The physiological data obtained were then analysed and elaborated by a dedicated algorithm available in the software (software V.6.1, algorithm V.2.2.3), and the metabolic data (total, active, and rest energy expenditure; metabolic equivalents of task (METs); total number of steps; physical activity duration; sleep duration; rest duration) collected. The software calculates the energy expenditure for each minute of data using complex pattern recognition algorithms, composed of ‘‘activity classification” (context detection) and ‘‘energy expenditure estimation” [23]. According to the manufacturer, the armband system accuracy is as follows: total calories/METs for free living activities, mean error <10%; total minutes of physical activity, mean error <5%; total step count, mean error <9%. The monitor is worn on the upper arm over the triceps muscle and at midhumerus point and is lightweight and comfortable enough to be used in the cave environment without hampering movement. All subjects were instructed to remove the armband only for bathing purposes (at about 200 m fom the cave’s entrance, a short sump must be passed to enter the inner part of the cave); when downloading the data, the software provided percentages of on-body time, confirming that all subjects weared the armband for at least 98% of the time they spent in the cave. Accelerometers are a practical and effective compromise between accuracy and feasibility for measuring energy expenditure. They are relatively inexpensive and generally well tolerated by research participants. The accuracy of SenseWear armbands in the measurement of energy expenditure both during daily life and exercise has been confirmed by several dedicated studies [25–27].

### Anthropometric measurements

Weight and height, upper arm, wrist, chest, waist, hip, thigh, ankle, and calf circumferences were taken by an experienced operator in accordance with standard international criteria [28]. Body mass index was calculated as weight/height2 (kg/m2).

### Nutritional status and dietary intake

Nutritional status of participants was assessed through bioelectric impedance (see below). Energy intake was determined by means of a self-administered dietary recall lasting four days, which included the day of cave activity. Reported data were analyzed through the Winfood® software.

### Bioimpedance analysis

The bioelectrical measurements (resistance, R, Ohm; reactance, Xc, Ohm) were taken according to standard international criteria [29], using the impedentiometer analyzer BIA 101 (Akern, Florence, Italy). Both the classic [30] and specific [31] bioelectrical vector analyses (BIVA) were applied. In classic BIVA, bioelectrical values are adjusted for height (R/H, Ohm/m; Xc/H, Ohm/m) in order to eliminate the conductor length effect, while in specific BIVA (R sp, Ohm cm; Xc sp, Ohm cm) they are adjusted for the whole conductor volume (correction factor A/L, in meters, where A (area) and L (length) are estimated as: A = (0.45 arm area + 0.10 waist area + 0.45 calf area) (m2); L = 1.1 H (m) and multiplied by a factor of 100. The phase angle is calculated as arctan Xc/R (degrees) in both BIVA approaches. Classic and specific vectors can be projected on a Cartesian plane defined by the adjusted resistance and reactance. Individual or sample characteristics can be compared with tolerance ellipses representing the variability of the reference population, thus allowing a semi-quantitative evaluation of body composition. In classic BIVA, the major axis of the tolerance ellipses refers to hydration status (dehydrated individuals towards the upper pole), while in specific BIVA it refers to the relative amount of fat mass. In both cases, the minor axis refers to body cell mass (higher values on the left side), particularly to muscular mass, and to extracellular-intracellular (ECW/ICW) water ratio (higher values on the right side). In this study, the reference population was represented by Italian adults [32] in classic BIVA and by Italo-Spanish young adults in specific BIVA [33]. The analyses of classic BIVA [34] and specific BIVA (http://specificbiva.unica.it/) were realized using freely available BIVA software.

### Statistical analysis

Weight and bioelectrical measurements taken before and after cave activity were compared using Student’s t test for paired samples. A Bayesian model of linear regression was applied [35] to estimate the effect of predictive variables on total energy expenditure (TEE; in log scale), considering the repetition of measurements in a normal day and in the day of cave activity (partial, i.e. limited to the activity in the cave, and full day, i.e. along the 24 hours). The Bayesian model is similar to an ordinary linear regression except that it provides the probability distribution of the coefficient, given the data, and it also allows for random individual effect, which are not possible with an ordinary linear regression. As every Bayesian model, it is estimated using prior distributions that are very weakly informative with respect to the sample size. Approximately, prior distributions employed in the research are as informative as just one observed subject. In this way, the inference is almost totally data driven and the prior has very little importance. Because of the large number of involved parameters the model has been estimated using Integrated Nested Laplace Approximation (INLA) [36] instead of the more usual Markov Chain Monte Carlo (MCMC) methods [35]. The regression included individual random effects in order to account for possible specific individual heterogeneity in the TEE which is not related to the predictive variables. Normality of the response variable was assessed either informally with a Quantile-Quantile plot as well as formally with the Kolmogorov-Smirnov test which did not reject the normality with a strong evidence in favor of it (p-value = 0.945). The effect of each variable is evaluated by calculating the exponential of the mean coefficient which corresponds to the increase or decrease in LOG in the TEE for a change in the predictive variable. An effect was considered significant if its posterior probability of being positive or negative was at least 95%. For example, if the coefficient of variable gender when it is M (men) has a 95% credible interval (i.e. the interval which contains the true value with 95% of probability) represented by an entirely positive range, the predicted variable TEE can be considered significantly higher in men than in women. The degree of the increment is estimated by the mean coefficient and the uncertainty by the 95% posterior credible interval. Positive values of the coefficient indicate a higher TEE value, while negative coefficient a reduced one. The set of predictors along with their interactions have been chosen according to Bayes Factors between the set of all possible involved models with all main effects and all two-by-two interactions. The goodness of the regression model has been empirically evaluated by comparing the observed data with those estimated by the model, considering the posterior credible interval of the predicted value of TEE. If such interval contains the observed value then it is possible to state that the case is adequately predicted by the model, otherwise not. The proportion of observed TEE adequately predicted by the model represents the goodness of fit measure of the regression model. This analysis avoids the use of p-values to assess the significance of the effects (i.e. predictors) in estimating the TEE, instead providing the probability of a significant effect and also its magnitude. Statistical analyses were performed using the free software R (http://www.R-project.org).

## Results

**Fig 3:**
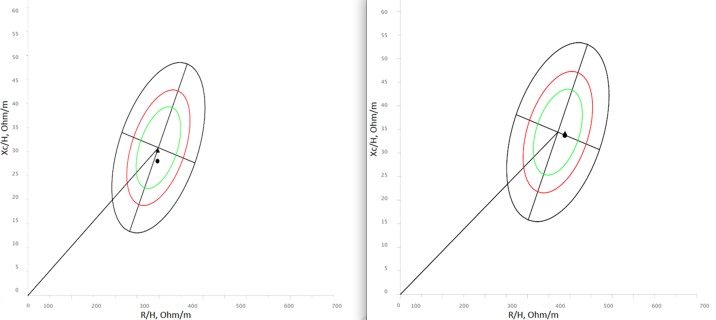
Individual bioelectrical vectors (specific BIVA). Individual bioelectrical vectors projected on the Italo-Spanish reference [33]. Men on the left, women on the right.

Anthropometric parameters of all 40 cavers involved in the study, and data concerning measured energy expenditure of the 36 cavers wearing metabolimeters during underground activity, are shown in Table 1. Results of the IET show that on average AT occurred at 163.9±12.5 w (i.e. about 74.5% of W_max_), while their W_max_ was 220.5±16.5 w. VO_2max_ was 2532.7±348.6 ml*min^-1^ (i.e. 34.6±3.4 ml/kg*min^-1^). These data indicated that cavers had a level of aerobic fitness higher than that of a sedentary population. Mean BMI was indicative of normal weight in both genders, even if a non-trivial proportion (50%) of men was overweight. However, cavers of both genders in general showed a quite low quantity of relative fat mass with respect to the reference sample of Italo-Spanish young adults, as measured through specific BIVA (Fig 3). Comparison of dietary intake and energy expenditure (TEE) in a normal day versus the full cave day and cave activity *per se*, offers interesting cues (Table 1). For both men and women, despite the fact that the intake during full cave day was significantly greater than an average normal day, energy expenditure during cave day largely exceeded the energy supplied by diet. Also, cave activity burned more energy than that present in food consumed underground. During full cave day, most of the intake was concentrated in the post-cave meal.

**Table 1:** Anthropometric measurements, physiological variables, and dietary intake.

	Men		Women	
	Mean	SD	Mean	SD
Height (m)	1.7	0.1	1.6	0.1
Weight (kg)	73.2	11.7	55.4	6.0
BMI	24.7	3.0	21.8	2.1
TEE (kcal/24h), normal day	3487.9	528.2	2367.3	316.6
TEE (kcal/24h), cave day	5128.5	862.5	3980.9	441.1
TEE (kcal/h), cave activity	287.5	48.5	225.4	27.9
MET’s, cave activity	4.1	0.7	4.1	0.5
Intake (kcal/24 h), normal day	2640.7	673.5	1858.1	324.3
Intake (kcal/24 h), cave day	3393.7	1530.3	2672.9	732.3
Intake (kcal/10 h), cave activity	1186.8	473.4	1008.2	513.2

BMI, body mass index; MET, metabolic equivalent of task; TEE, total energy expenditure; SD, standard deviation.

**Fig 4:**
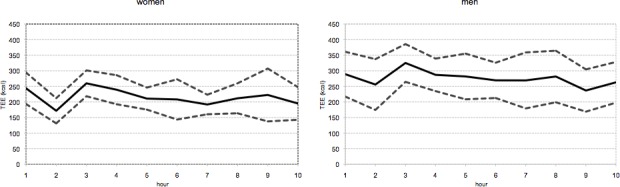
Time course of total energy expenditure (TEE, kcal/h) during cave activity, for women and men. Dotted lines show confidence interval.

The time course of total energy expenditure (TEE, kcal/h) for women and men during caving activity is shown in Fig 4. The graph gives a precise idea of the energy required by all subjects to sustain cave activity, and shows for both women and men a rather constant energetic effort along the entire 10 hours span of underground exploration. The decrease in energy expenditure notable at two hours after cave entrance, followed by a rapid increase in energy expenditure at three hours after cave entrance, are probably due to the particular morphology of Su Palu cave, and the succession of technical pauses (e.g., passage of sump) and climbing/rope work. Most likely, initial acclimatization to cave environment also played a role in shaping the time course of energy expenditure [37], but more focused experimental work should be conducted to ascertain this.

**Fig 5:**
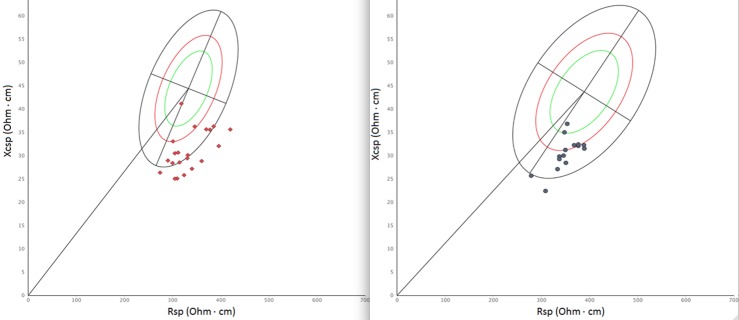
Mean bioelectrical vectors (classic BIVA). Mean bioelectrical values projected on the Italian reference [32]. Men on the left, women on the right.

During the day of cave activity, weight (Student’s t = 2.4; p < 0.05), classic and specific reactance (Student’s t = -2.6 in both cases; p < 0.05), and phase angle (Student’s t = -2.9; p < 0.05) increased significantly in men, but not in women (Table 2). The lack of significant variation in specific resistance indicates that the percentage of fat mass remained quite constant [31]. As for hydration status, classic BIVA showed that cavers were within the range of normal hydration before and after the cave activity (Fig 5). However, the bioelectrical change observed in men indicates a reduction of extracellular water [31].

**Table 2:** Bioelectrical values before (pre) and after (post) partial day cave activity.

	Pre-cave activity		Post-cave activity		
	Mean	SD	Mean	SD	p
Men					
R/H	296.6	38.5	297.5	33.1	
Xc/H	28.1	5.9	30.3	6.2	*
Rsp	335.7	38.3	338.5	42.3	
Xcsp	31.5	5.0	34.0	5.5	*
Phase angle	9.4	1.3	10.1	1.7	**
Women					
R/H	388.6	34.1	389.2	36.8	
Xc/H	33.7	3.2	34.2	4.7	
Rsp	349.5	29.5	349.9	30.4	
Xcsp	30.4	3.6	30.8	4.4	
Phase angle	8.7	0.8	8.8	1.0	

H, height; R, resistance; Rsp, specific resistance; Xc, reactance; Xcsp: specific reactance

* p < 0.05

** p < 0.01; SD, standard deviation.

**Fig 6:**
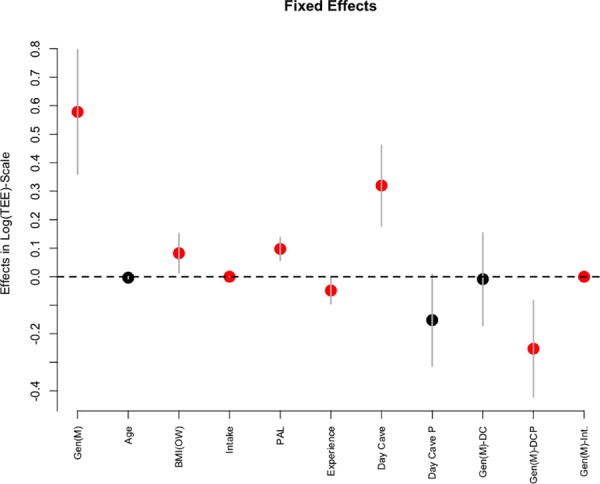
Bayesan linear regression estimate of the effect of predictive variables on total energy expenditure. Mean effects (bullets) along with 95% posterior credible intervals. Significant effects are in red. GEN (M), gender (men); BMI (OW), BMI class (overweight); PAL, Physical Activity Level; Day cave P, Day cave, partial; GEN (M)–DC, gender (men)—Day cave; GEN (M)–DCP, gender (men)—Day cave, partial; GEN (M)–INT, gender (men)–intake.

The regression model allowed the correct estimate of 62% of the observations. Of the eight variables considered in the regression along with three interactions, five variables–gender, physical status, intake, physical activity level (PAL), cave activity–significantly predicted TEE, together with the interaction among gender and cave activity (partial), and among gender and intake (Table 3, Fig 6). In particular, not surprisingly men showed a TEE higher than that of women, with a mean value of 1.79, that is 79% more TEE than women. Overweight individuals showed 8% more TEE than normal weight ones, while age was not significantly related to TEE. Experienced cavers had a 5% lower TEE than the less skilled ones. Full day cave activity induced a significantly larger effect than normal day activity (32% more TEE), while partial day cave activity did not show a larger TEE with respect to the normal full day. Men showed a significantly lower TEE (around 22% less) than women during cave activity, as shown by the significant interaction between gender and partial day activity. In other terms, women required comparatively more energetic efforts than men during underground activity to perform the same task. The intake and PAL were both significantly related to TEE (11% and 0.01% of increase of TEE, respectively). Intake also showed a significant interaction with gender in predicting TEE, as in men the increase of TEE was slightly but significantly lower than women (1/10000 less).

**Table 3:** Posterior distribution of effects along with posterior credible intervals.

	Mean	SD	95% Credible Interval	Statistical significance
Gender (men)	0.58	0.11	(0.36, 0.80)	yes
Age	-0.03e^-1^	0.02e^-1^	(-0.07e^-1^, 0.06e^-2^)	no
BMI class (overweight)	0.08	0.03	(0.01, 0.15)	yes
Intake (cave activity)	0.01e^-2^	0.00	(0.00, 0.02e^-2^)	yes
PAL	0.10	0.02	(0.06, 0.14)	yes
Experience	-0.05	0.02	(-0.09, -0.02e^-1^)	yes
Day cave	0.32	0.07	(0.18, 0.46)	yes
Day cave, partial	-0.15	0.08	(-0.31, 0.08^e-1^)	no
Gender (men)—intake	-0.01e^-2^	0.00	(-0.02e^-2^, 0.00)	yes
Gender (men)—day cave	-0.08e^-1^	0.08	(-0.17, 0.15)	no
Gender (men)—day cave, partial	-0.25	0.09	(-0.42, -0.08)	yes

Significant effects are those for which the respective posterior credible intervals do not contain 0; BMI, body mass index; PAL, physical activity level; SD, standard deviation. For more details, see Methods.

## Discussion

This is the first study to measure energy expenditure during cave exploration. Whether caving should be regarded as a sport or rather a recreational physical activity is a matter of discussion. Historically, caving can be considered a descendant of mountaineering (alpinism) and climbing, and most of the caving techniques and equipment derive directly from those used in those disciplines. Climbing (climbing wall) will be on display as a new sport at the Buenos Aires Youth Olympic Games in 2018, a teaser of what is to come in 2020 at the Olympic Games in Tokyo [38]. On the other hand, what certainly is lacking in caving to be fully defined as a sport is that it does not include any element of competition [39]. We opt for considering caving as a recreational physical activity.

Comparing our recorded data on energy expenditure during caving with published surveys of the intensity of physical activity in sports [40,41] and guidelines on the definition of the level of intensity of general physical activities [42], it emerges that caving can be considered a moderate intensity exercise. Although caving is sometimes resembled to alpinism and climbing, as explained above, both rock ascending and rappelling are much more energy intense [40,41,43]. When non-competitive, recreational physical activities are taken as a reference, the exercise intensity required by caving sits in an intermediate position between, say, recreational visits to natural environments (≈3–3,5 METs) [44] and recreational scuba diving (≈5–6 METs) [45]. However, since cave explorations are usually carried out for a prolonged time, cavers must often sustain an overall elevated energy expenditure and thus might well experience a considerable increase in fatigue, which in turn can increase the risk of injuries. Great care should therefore be paid to planning underground activities taking into proper consideration the physical condition and experience of involved cavers. As our study shows, experienced cavers have a lower mean energy expenditure than less skilled ones, and can therefore sustain more extensive underground explorations. Thus, experienced cavers have a higher yield (work/energy expenditure), i.e. consume less energy for doing the same thing everyone else is doing: a well known fact in biomechanics [46]. However, while optimized efficiency–intended as minimized energy expenditure and maximized work output–is evident for experienced cavers versus less experienced ones, it not clear at this stage whether these results can be achieved only through extensive frequentation of caves or if a specific outdoor/indoor training program, designed to closely reproduce the type of physical activity cavers will be performing during cave exploration, would have comparable effects on efficiency during caving activity [47,48].

We determined the nutritional habits of cavers, both during a normal day and during cave activity. In general terms, the main purpose of nutrition is to ensure the compensation of increased energy consumption and the need for nutrients in the subject’s body, thereby enabling maximum adaptation to physical loads and also to reduce dehydration and further sustain the activity. Our survey indicates that, while underground, cavers tend to introduce less calories than those burned during cave activity. Subjects had a cave energy intake in the range 1000–1200 kcal, while TEE was in the range 225–287 kcal/h, for 8–10 hours. Cavers consumed light-weight, physically tough, high-energy-dense foods, such as dried fruits, nuts, chocolate, energetic bars, parmesan cheese, and honey. Such dietary choices are in line with the necessity of avoiding heavy loads to be carried over long distances during cave exploration and the inclination to stick to easy-to-digest foods during exercise [49]. Such a large discrepancy between energy intake and energy expenditure is probably sustainable in the case of cave explorations lasting up to 15–16 hours. However, for longer underground explorations, a more balanced diet is required, with food portions high in carbohydrates, low in fat, and adequate in protein, able to fully restore lost calories. Clearly, energy intake is an important variable to be considered when devising strategies for optimizing performance, even in recreational physical activities such as caving. However, our survey showed that cavers did not adopt any particular nutritional strategy during the days before cave activity, not complying specifically with any of the available recommendations on energy intake before, during and after exercise [50]. As mentioned above, the selection of foods consumed during cave activity was merely based on previous experience and practicality, rather than an estimation of energy intake needs and subsequent preparation of adequate food portions. Although poor compliance with official nutritional recommendations is not rare among athletes [51], in the case of caving the lack of evidence-based knowledge on nutritional requirements for this activity makes the problem more radical, and calls for tailored studies aimed to drafting guidelines for an adequate nutrition pre- and during-cave activity, to improve performance and reduce the risk of incidents.

Hydration is an important physiological parameter for subjects performing exercise in general. In particular, hypohydration and electrolyte balance perturbations can impair aerobic exercise performance, this effect being closely related to environmental parameters like heat, cold, and air humidity [52]. In the case of cavers, it is especially intriguing to monitor fluid balance and body water, given the long-duration activity and the reduced tendency to drink in the underground environment because of high air humidity. As skin temperature is elevated in proportion to ambient temperature and humidity, and high skin temperature is in turn linked to an increased requirement on sweat secretion and evaporation to regulate body temperature [52], the possibility of hypohydration (mainly due to sweat loss) during caving is concrete. BIVA analysis indicated a normal overall level of hydration for cavers, but a tendency to the reduction of extracellular water in men. This suggests that a more prolonged, or physically demanding cave activity might ensue in hypo-osmolal dehydration, if an appropriate amount of salts is not introduced together with water [53]. Ingestion of fluids is effective in limiting the detrimental effects on performance but while in our case cavers only drank salt-free water (max. 1 L per person), it might be recommendable to make use of thirst quenchers able to replace the electrolytes lost with sweat, in addition to water.

As caves vary greatly in shape, length, and environmental conditions (relative air humidity and temperature), it is not possible to translate our results to all caving activities in a straightforward manner. For example, it would be of great interest to measure energy expenditure and variations of body composition (hydration) in subjects involved in explorations of caves in tropical settings, where air temperature can easily attain 35°C [54]. In these cases, indeed, the combination of hot air and humidity would probably lead to a heat load that would impact heavily on hydration and exercise efficiency [37]. Also, not all cavers perform the same activities. For example, cave rescue teams might be called to sustain heavy work loads underground for many hours, sometimes days, to complete rescue operations [55]. Finally, caves have lately been selected as natural environments apt to train astronauts for long periods of permanence in space and extraterrestrial exploration. This because “caves are dark, remote places, with constant temperature, many logistic problems and stressors (isolation, communication and supply difficulties, physical barriers), and their exploration requires discipline, teamwork, technical skills and a great deal of behavioural adaptation,” [56]. In all these cases a deep knowledge of physical adaptation to exercise and/or permanence in cave environments and associated changes in body composition and nutritional requirements, as preliminarily assessed by our study, would be crucial in order to achieve better performances and reduce the risk of injury.

## Conclusions

In conclusion, we have provided evidence that, despite inherent logistic difficulties, energy expenditure and modification of physiological parameters and body composition during caving can be measured and interpreted. Also, nutritional habits and hydration of cavers were assessed, revealing interesting details that will be useful in defining an ideal diet for this special type of physical activity that requires a protracted, although moderately intense, exercise under singular environmental conditions. Since the scientific literature on caving is scarce, its distinguishing characteristics call for more research to better determine the physiological basis of this activity in a variety of settings and for multiple purposes. From a methodological point of view, we have shown the usefulness and suitability of bioimpedance vector analysis to evaluate body composition variations in subjects performing physical activity in extreme environments, such as those related to cave exploration.
