# Correction: Energy expenditure in caving

**DOI:** 10.1371/journal.pone.0196028

**Published:** 2018-04-13

**Authors:** Giorgia Antoni, Elisabetta Marini, Nicoletta Curreli, Valerio Tuveri, Ornella Comandini, Stefano Cabras, Silvia Gabba, Clelia Madeddu, Antonio Crisafulli, Andrea C. Rinaldi

The images for Figs 3 and 5 are incorrectly switched. The image that appears as Fig 3 should be Fig 5, and the image that appears as Fig 5 should be Fig 3. The figure captions appear in the correct order.

**Fig 3:**
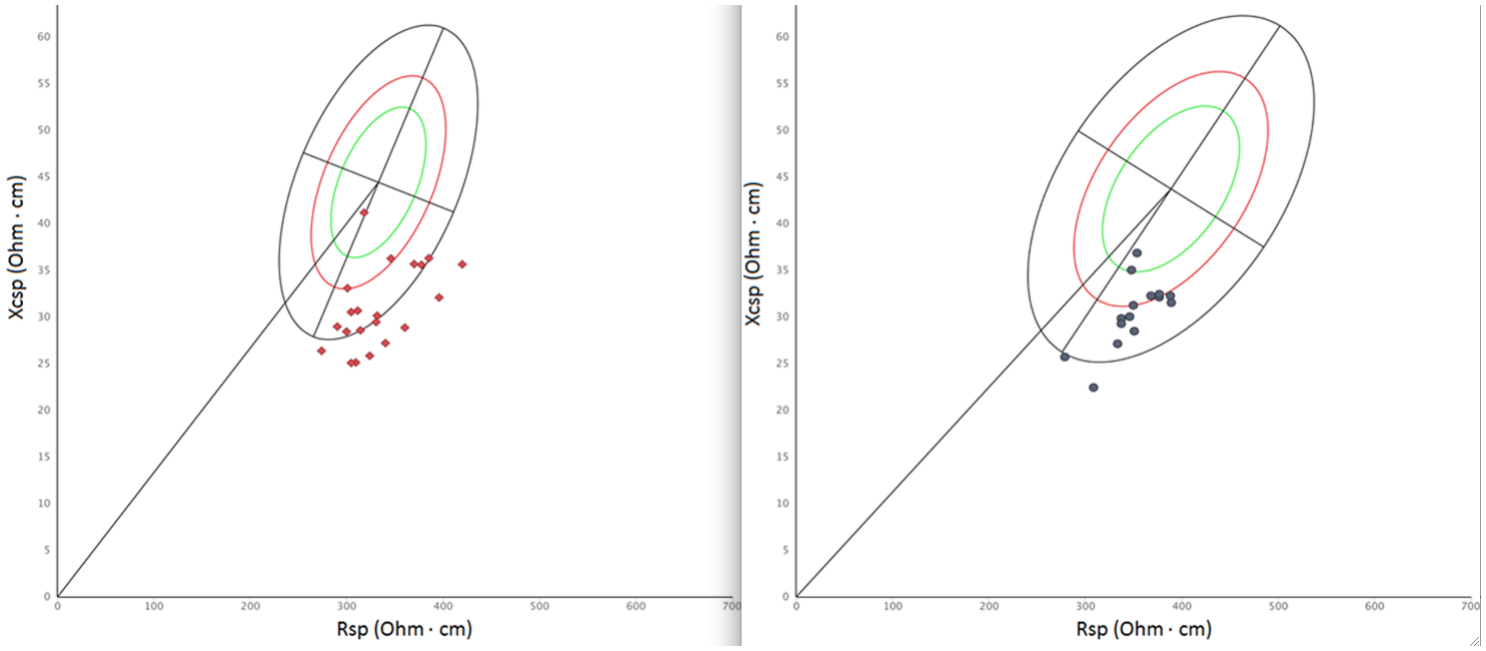
Individual bioelectrical vectors (specific BIVA). Individual bioelectrical vectors projected on the Italo-Spanish reference [33]. Men on the left, women on the right.

**Fig 5:**
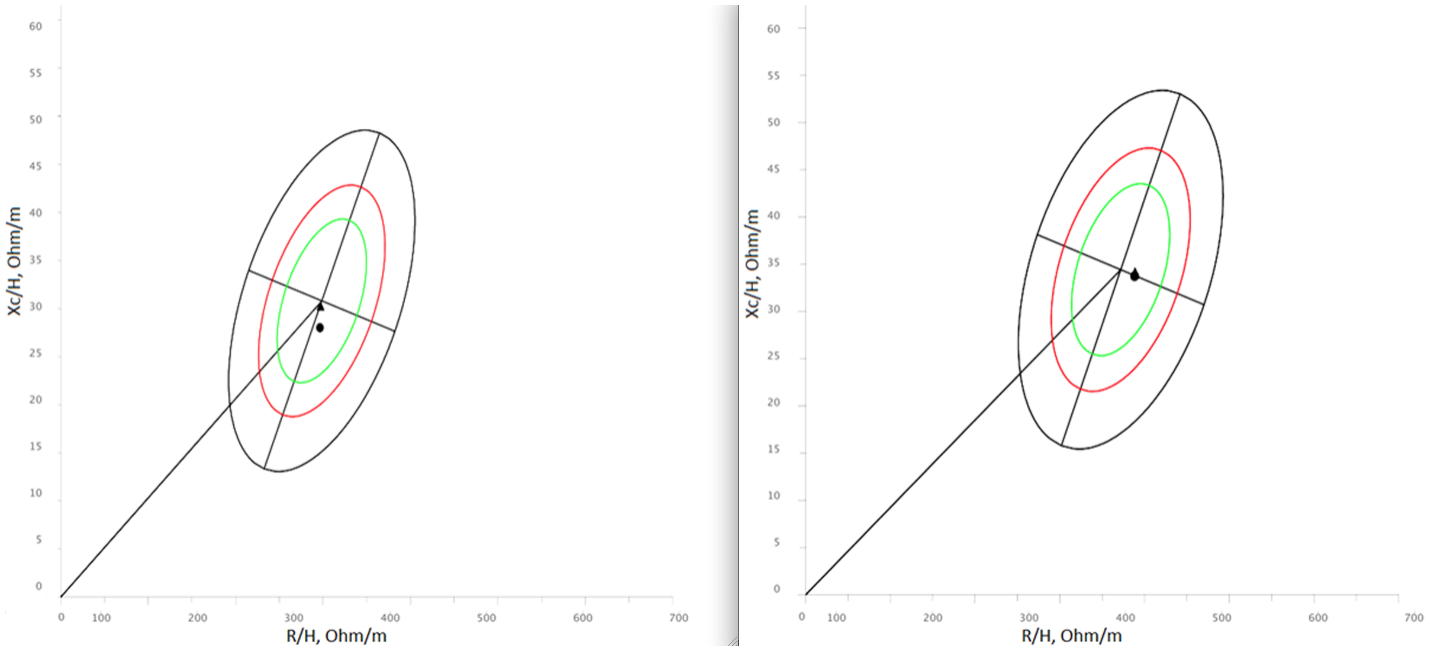
Mean bioelectrical vectors (classic BIVA). Mean bioelectrical values projected on the Italian reference [32]. Men on the left, women on the right.
